# Decision-Making Teaching Practice Based on the Maximum Entropy Method in a Water Engineering Economics Course

**DOI:** 10.3390/e25030441

**Published:** 2023-03-02

**Authors:** Runjuan Zhou, Yingke Sun, Shuai Shao, Kuo Zhang, Ming Zhang

**Affiliations:** 1 School of Chemical and Environmental Engineering, Anhui Polytechnic University, Wuhu 241000, China; 2 School of Civil Engineering, Anhui Polytechnic University, Wuhu 241000, China

**Keywords:** decision-making, teaching, water engineering economics, maximum entropy, genetic algorithm

## Abstract

The purpose of this paper is to put forward a decision model with wide applicability and differentiated decision scheme scores so as to improve the ability of students to learn during a water engineering economics course. The main novelty and contributions of this paper are that the multi-attribute decision-making method proposed is more objective and does not require rich subjective experience from decision-makers in the application process, which is particularly suitable for beginners who are learning in a water engineering economics course. The method involves standardizing each index value of the decision scheme first, constructing the objective function of maximum entropy distribution, calculating the weight of each index by the genetic algorithm, and finally ranking the pros and cons of the scheme according to the score of each scheme. The example results of three water engineering scheme decisions show that the maximum entropy model proposed in this paper can achieve reasonable decision results, and there is a large degree of differentiation between the decision schemes. The proposed scheme, a decision maximum entropy model, has wide applicability, can improve the rationality of the decisions made regarding water engineering schemes, and can be popularized and applied when teaching decision-making in water engineering economics courses.

## 1. Introduction

Water engineering economics is an important course that involves studying decision-making and evaluation in the context of water resources, environmental engineering, and other engineering projects [1,2]. In the traditional teaching mode, teachers and students have formed relatively fixed teaching and assessment methods and content in order to satisfy the requirements of the school’s assessment system [3,4]. In this system, the teachers use the same textbook to select content. Therefore, in the classroom, teachers repeat similar content year after year [5]. Under this system, the contents of students’ assessments are relatively fixed, and the examination review materials, such as question banks, are formed year after year. As a result, evaluating students’ learning outcomes becomes more difficult, and students also become more indifferent to the content of teachers’ classes [6,7]. In view of this deficiency, Oliver-Hoyo [8], Griffiths [9], Rijst [10], and others have carried out, in succession, studies on the nexus of scientific research and teaching. These research results show that the research-teaching nexus has many benefits, including being able to provide a new development model for China’s current higher education and a new training model for upcoming talents in systematic research [11]. In addition, China’s *14th Five-Year Plan for Water Science and Technology Innovation* also proposes leading the new phase of Chinese high-quality water conservancy development with a high level of scientific and technological innovation and support [12]. In order to accomplish this task, several important and immediate problems must be solved, including how to improve the practical ability of students and how to strengthen the study of professional courses [13]. Applying “the research-teaching nexus” in teaching practice causes students to no longer be blindly superstitious about textbooks. It also enables students to acquire new knowledge. This teaching mode has an essential enlightening effect on cultivating students’ ability to innovate to some extent.

Water engineering economics courses involve a wide range of subjects, among which the multi-attribute decision-making problem has yielded a large number of research results in academic circles [14,15]. However, some of the above results have caused a great deal of trouble for teachers and students regarding teaching practices because of a lack of adaptability and differentiation. In addition, current teaching processes still adopt the research results of decades before, thus resulting in the processes seriously lagging behind current developments [16,17,18]. The traditional teaching model and decision-making model make it difficult for students to acquire corresponding decision-making abilities. In the course of water engineering economics, the multi-attribute decision-making problem is generally an uncertain decision-making problem with unknown attribute weight, which is of great significance in the theory and practice of system engineering. Whether a decision is right or not is often related to the success or failure of an individual’s career and a great gain or loss of interest [19,20]. Owing to the different decision criteria adopted by decision-makers, the ranking results of decision schemes are often unreasonable and not convenient for decision-makers to use in order to make the best decisions [21,22]. In recent years, researchers have established a variety of models—including the analytic hierarchy process optimization model [23], the grey comprehensive optimization model [24], the fuzzy comprehensive optimization model [25], the artificial neural network optimization model [25], etc.—which have played a positive role in the optimization of water engineering schemes. However, scheme selection involves numerous decision indicators, including different dimensions of each indicator, and it is often difficult to determine the weight of each indicator. There are certain difficulties in the practical application of these methods, such as the fact that the differences in the comprehensive score value of each scheme are not obvious; additionally, the model results are affected by subjective factors. In particular, most traditional decision-making methods need to use the rich subjective experience of decision-makers. This is important as beginners in water engineering economics courses often do not have such experience. Therefore, in order to fill this gap, the novelty of our work is mainly that we propose a more objective multi-attribute decision-making method and then apply it to the teaching of water engineering economics.

Generalized entropy is the expansion of the information entropy theory, which can effectively measure the uncertainty of weight distribution. In a case where only part of the information is available, the probability distribution that meets the constraint conditions, and has the maximum entropy, should be taken when making an inference about the weight distribution of various indicators. With greater entropy, fewer constraints and assumptions are added artificially, and the deviation of the calculated probability distribution is smaller [26,27,28]. For an uncertain decision-making problem, in order to obtain the score value of the scheme, it is necessary to satisfy the maximum entropy of the weight distribution of each index. It is also necessary to ensure that the score value of each decision-making scheme has a certain degree of differentiation, and that it is dispersed as far as possible when only the decision matrix values of various schemes, under various indicators, are mastered. Therefore, a complex optimization problem is constructed with the function of the decision matrix as the objective function and the weight of each index as the optimization variable.

Based on the above analysis, while using the known information of the decision matrix, the objective function of the maximum entropy of the indexed weight distribution and the maximum variance of the score values of the resulting decisions are constructed. Then, the probability distribution of each index weight is optimized and deduced using optimization algorithms, such as the accelerated genetic algorithm, to transform the uncertain decision problem into a risk-type decision analysis problem for the purposes of decision analysis. The above decision-making process is known as the maximum entropy method (MEM for short) in the context of water engineering project decision-making.

Holding all the above points in mind, the primary objectives of the work are the following:

(1) To put forward a decision method for the teaching of water engineering economics. This method should be widely applicable to all kinds of water engineering scheme decision problems;
(2) To establish a decision analysis method that can simultaneously solve the weight distribution of the decision index and the score value of the decision scheme. Further, the score value of the decision scheme should have a certain degree of differentiation;
(3) To avoid adding redundant subjective information, the maximum entropy distribution is taken as the index weight distribution, which can improve the objectivity of the decision-making model;
(4) To give some examples to understand the feasibility, reliability, and effectiveness of this study, three decision-making examples in water engineering are applied to the case study in order to show their superiority and advantages.

The rest of the manuscript is organized as follows: In Section 2, some basic concepts of the existing sets are reviewed, and the objective function and solution framework of the MEM model are established. In Section 3, we present three cases, all of which are commonly used in the teaching of water engineering economics, among which the first is the optimization of the water-saving scheme, the second is the decision of urban water supply engineering scheme, and the third is the decision of water resource allocation scheme. In these cases, we compare and analyze the index weight, scheme score value, and scheme ranking, respectively. Finally, Section 4 concludes the paper.

## 2. Materials and Methods

### 2.1. Maximum Entropy Method

For a multi-attribute decision-making problem, it is generally assumed that there are *n* decision schemes $S_{i}\left(i=1\sim n\right)$ and *m* decision indicators, which constitute the decision matrix $X={\left(X_{ij}\right)}_{n*m}$ of the water engineering project scheme. The establishment of the MEM model includes four steps: preprocessing of the decision matrix data, construction of the objective function, optimization of the solution, and forming of the decision scheme.

Step 1: Data preprocessing.

In order to facilitate unified processing of each index value in each decision matrix, it is necessary to convert it into an index that changes in the same direction.

The case where bigger values of an indicator are better can be expressed by Equation (1) [26].

(1) $$r_{ij}=\frac{x_{ij}-\min\left(x_{i}\right)}{\max\left(x_{i}\right)-\min\left(x_{i}\right)}$$

The case where smaller values of an indicator are better can be expressed by Equation (2) [26].

(2) $$r_{ij}=\frac{\max\left(x_{i}\right)-x_{ij}}{\max\left(x_{i}\right)-\min\left(x_{i}\right)}$$

The intermediate optimal indicators can be expressed by Equation (3) [26]:

(3) $$r_{ij}=1-\frac{\left|x_{lj}-x_{ij}\right|}{\max\left|x_{lj}-x_{ij}\right|}$$

where $r_{ij}$ is the index value of each scheme after uniformity processing, i=1~n, j=1~m; $x_{ij}$ is the original index value; and$\;x_{lj}$ is the ideal value of the index of the more intermediate and better type.

Step 2: Construct the objective function.

Let the probability distribution of the weight of *m* indicators be $W={\left(w_{j}\right)}_{m}$; then, the entropy *H* of each indicator can be calculated as Equation (4) [29,30]:

(4) $$H=-\overset{m}{\underset{j=1}{\sum }}w_{j}\log\left(w_{j}\right)$$

where the probability distribution $w_{j}$ of each index should ensure the normalization of the constraint Equation (5).

(5) $$\overset{m}{\underset{j=1}{\sum }}w_{j}=1;\;w_{j}\geq 0,\;j=1\sim m$$

Depending on the principle of maximum entropy [29,30], the probability distribution is the most objective and the deviation is the least when the entropy of the index weight probability distribution is the maximum. Based on the decision matrix $X={\left(X_{ij}\right)}_{n*m}$, the score value $y_{i}$ of the decision scheme $S_{i}$ under the weight of each index is calculated, as shown in Equation (6).

(6) $$y_{i}=\overset{m}{\underset{j=1}{\sum }}w_{j}x_{ij},\;i=1\sim n$$

In order to make the decision results of each scheme easy to distinguish, it is necessary to render the score values of each decision scheme to be as dispersed as possible, such that the standard deviation *V* of the score value is at the maximum, as shown in Equation (7) [26]:

(7) $$\max V=\sqrt{\frac{1}{n}\overset{n}{\underset{i=1}{\sum }}{\left(y_{i}-\bar{y}\right)}^{2}}$$

where $\bar{y}$ is the average score value of each decision scheme, as shown in Equation (8).

(8) $$\bar{y}=\frac{1}{n}\overset{n}{\underset{i=1}{\sum }}y_{i}$$

From the above analysis, it can be seen that the decision-making problem in water engineering economics teaching can be solved by solving the optimization problem, such as is found in Equation (9) [26], to obtain the weight distribution of each index, and then to convert it into a more manageable risk-type decision-making problem for program decision-making.

(9) $$\begin{array}{c} \max\;Q\left(w_{j}\right)=H*V\\ \;\;\;\;\;\;\;\;\;\;\;\;\;\;\;\;\;\;\;\;\;\;\;\;\;s.t.\;\;\overset{m}{\underset{j=1}{\sum }}w_{j}=1,\;w_{j}\geq 0,j=1\sim m \end{array}$$

Step 3: Optimize the solution on the basis of the genetic algorithm.

Equation (9) is a multivariable, constrained, nonlinear optimization problem, which can be solved by an accelerated genetic algorithm [31,32] that simulates the survival of the fittest rule and the mechanism of chromosome exchange within a population. After initialization, the fitness value calculation, selection, crossover, and mutation genetic operations are performed. Then, the evolution and acceleration operations to complete the accelerated genetic algorithm optimization process are conducted. The index weight distribution $W={\left(w_{j}\right)}_{m}$ obtained by optimization Equation (9) is the distribution with the least uncertainty in all of the solution space.

Step 4: Decision and analysis of the water engineering scheme.

The weight distribution of each index is calculated by an optimization technique, and the uncertain decision problem with an unknown original weight is transformed into a risk-type decision problem. The weight distribution of each index $W={\left(w_{j}\right)}_{m}$ is added into Equation (6) to obtain the score value of each decision scheme, and the decision scheme with the highest ranking is the most optimal scheme.

When we solve the multi-attribute decision-making problem with multiple schemes, we generally hope that the score values of the decision schemes can be well distinguished to avoid the situation that the score results of several decision schemes are exactly equal or are very close to one another. This situation will confuse the decision-makers, and they do not know how make the right and most reasonable decision. In order to verify the differentiation, rationality, and operability of the score value of the MEM decision scheme, we also compare the traditional decision methods in decision research, such as the projection pursuit method [33], the analytic hierarchy process [23], the grey relational degree method [24], etc.

**Figure 1:**
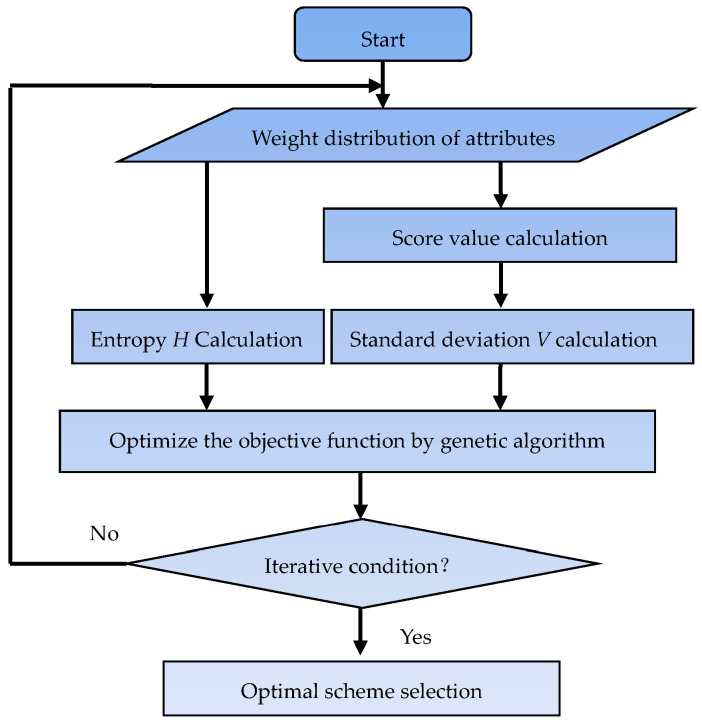
The flow chart of the maximum entropy method.

The flow chart of the maximum entropy decision model with an indefinite weight is shown in Figure 1.

The iteration in Figure 1 is the main process of solving Equation (9) via the genetic algorithm. Since the weight information is unknown, the initial value of the attribute weight is generated by a random number generation program at the beginning of calculation. Then, the entropy is calculated by Equation (4). In addition, the score value and standard deviation of each scheme is calculated by Equations (6) and (7). Then, the value of the objective function is calculated by Equation (9). According to the principle of genetic algorithms, when the value of the objective function of a certain number of times does not increase, the calculation stops; otherwise, the optimization generates a new attribute weight, and the above calculation process is repeated until the iteration stop condition is reached. At this time, the weight that is obtained is considered as the weight of each decision indicator and can be used for the next step of the scheme decision.

### 2.2. Materials of Three Examples in the Teaching of Water Engineering Economics

Water engineering scheme optimization is a typical multi-index scheme optimization problem. In this paper, three cases of engineering, including water-saving irrigation, water supply engineering, and water resources allocation, are selected. Moreover, the results of other decision-making methods are compared to verify the rationality of the maximum entropy decision-making method.

#### 2.2.1. Example 1—Optimization of the Water-Saving Irrigation Scheme

The optimization of the water-saving irrigation scheme is an important research subject in water engineering economics courses. Furthermore, it involves many concepts and methods in its application, such as a national economic evaluation, technical evaluation, internal rate of return, net present value, benefit–cost ratio, and payback period.

This paper takes the water-saving irrigation project that is constructed in the literature [23] as an example to carry out a comparative study on a scheme optimization based on MEM. There are four known water-saving irrigation projects to choose from (pipeline irrigation, sprinkler irrigation, drip irrigation, and tubular outflow irrigation) (schemes S1~S4). The decision indicators of these projects are shown in Table 1. Each index is as follows, X1: NPV, net present value (CNY ten thousand); X2: RR, rate of return (%); X3: PP, payback period (a); X4: BCR, benefit–cost ratio (-); X5: IU, irrigation uniformity (%); X6: II, intensity of irrigation (mm•h^−1^); X7: WUR, water utilization rate (%); X8: SR, safety and reliability (%); X9: CA, crops adaptability (%); X10: PF, popularity among farmers (%); and X11: CC, convenience of construction (%).

#### 2.2.2. Example 2—Urban Water Supply Scheme Optimization

Urban water supply scheme optimization is the process of selecting the relative best scheme from the inadequate water supply schemes. It is one of the knowledge areas that students who are majoring in water supply and drainage should master. Moreover, it is also one of the more common application cases in water engineering economics courses. This paper takes the Yellow River Diversion water supply project in Longhu Zhengdong New District, China as an example to carry out a case study of the MEM. The water supply process is an important measure for the construction of ecological Zhengzhou, and is also an important supporting construction project of the Longhu water system in the Zhengdong New District. In this paper, the MEM model is used to comprehensively evaluate the schemes of the Yellow River Diversion water supply project. In addition, the best scheme for the water supply project is selected.

According to the actual situation of the project, six water supply schemes are proposed in the literature [24], which are as follows: the Madu fully concealed pipe scheme (S1); the Huayuankou open Channel Scheme (S2); the Huayuankou canal and pipe combination scheme (S3); the Gangli Full Open Channel Scheme (S4); the Gangli canal combination scheme (S5); and the Mangshan Drainage channel Integration Scheme (S6). The indexes in the scheme are, X1: LWTL—length of water transmission line (km); X2: PI—project investment (CNY ten thousand); X3: AOC—annual operating fee (CNY ten thousand); X4: PP—payback period (a); X5: IFCYR—impact of flood control on the Yellow River (-); X6: ROP—reuse of original project (-); X7: SWIC—superiority of the water intake condition (-); X8: OMD—operation management difficulty(-); X9: WQGD—water quality assurance degree (-); and X10: EEID—ecological environment improvement degree (-). The schemes and index values for the aforementioned are given in Table 2.

#### 2.2.3. Example 3—Water Resource Allocation Scheme Decision

Water resources allocation refers to the allocation of a variety of available water sources between regions and water departments within a basin or region. This scheme follows the principles of high efficiency, fairness, and sustainability, while using various engineering and non-engineering measures to reasonably curb demand, effectively increase water supply, and to actively protect the ecological environment. The decision that is to be made with respect to the water resources allocation scheme is a necessary means to seek a reasonable, technical, and economic scheme for a water conservancy project. Further, it is also an important way to guarantee project quality, control project investment, and to reduce operation cost. It involves the consideration of many factors, such as technology, economy, environment, resources, society, and security. At present, subjective analysis methods, such as the grading method and analytic hierarchy process (AHP), are typically used in the teaching practice of water engineering economics, which already lags far behind the development speed of decision theory.

In Example 3, a water resources allocation project in Tianjin, China—which is mentioned in the literature [25]—is taken as an example, and the proposed MEM method is implemented in order to carry out a comparative analysis. According to the situation there regarding social and economic development and water conservancy project development in Tianjin, China, eight water resources allocation schemes S1-S8 with good representativeness and strong feasibility (based on the three principles of representativeness of water demand, representativeness of water supply, and representativeness of project layout in water resources allocation through analysis, comparison, and screening) are selected after abandoning those that were evidently inferior schemes.

The evaluation indexes are established from the social rationality, economic rationality, resource rationality, and efficiency rationality of water resources, among which the social rationality indexes include, X1: RWLR—regional water lacking rate (%); X2: IWLR—industrial water lacking rate (%); and X3: AWLR—agricultural water lacking rate (%). The economic rationality indexes include, X4: IOPCMW—industrial output per cubic meter of water (CNY ten thousand/m^3^); X5: GRIAV—growth rate of the industrial added value (%); X6: IWP—investment into the water project (CNY hundred million). The resource rationality indexes include X7: sewage recycling amount (hundred million m^3^). The efficiency rationality index includes, X8: IWRR—industrial water reuse rate (%); X9: UCIA—utilization coefficient of agricultural irrigation (-); and X10: UWSPLR—urban water supply network leakage rate (%). The index values of each scheme are given in Table 3.

## 3. Results and Discussion

### 3.1. Example 1

#### 3.1.1. Calculation Result of Index Weight in Example 1

**Figure 2:**
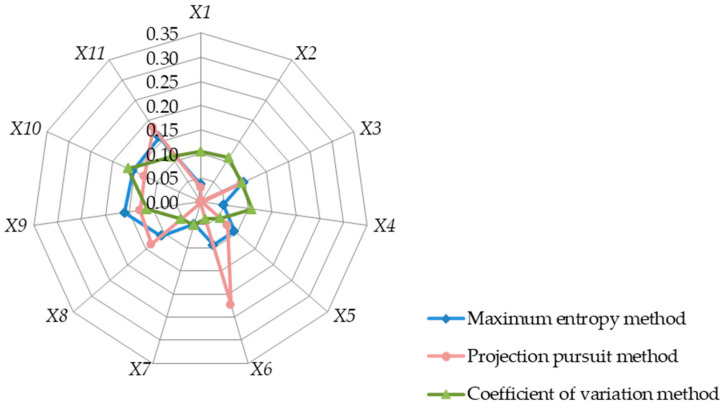
Radar map of the index weights of each method for Example 1.

The genetic algorithm is adopted to calculate the weight *w_j_* of each indicator according to the process in Figure 1, as shown in Table 4. For the convenience of comparison, the index weight of the objective weighting method pursuit projection (PP for short) [33,34] and the coefficient of variation (CV for short) [24,35] are also listed in Table 4. Among them, the weight of the PP is the result that is found in the literature [33], and the weight of the CV is calculated according to the literature [24,34]. In order to show the weight distribution more clearly, the radar map of each indicator is drawn in Figure 2.

As shown in Figure 2, the weight calculated by the proposed MEM method is consistent with the change trend of the PP method and the CV method on the whole. The weight of each index changes around 0.1, and a few of the weight values have big differences. Table 4 demonstrates that the weight calculated by the PP method shows obvious extremes and certain groupings. For example, the maximum weight of the X6 index is 0.223, while that of X8~X9 is about 0.1. The other indicators, such as X2, X4, and X7, are concentrated around 0.002. The weight calculated by the CV method presents two categories. The first category is around 0.1 for X1~X4, X9, and X10, whereas the other category is approximately 0.05 for X5~X8. 

The weight calculation results of the MEM take into account the PP method and the coefficient of variation, in which the weight of the “Intensity of irrigation” of the X6 index is calculated as 0.094, which is more consistent with the actual situation of the water-saving irrigation scheme decision. The weight of the “Rate of return” of the X2 index in the results of the MEM and PP method is relatively small, among which the weight of the MEM is only 0.000088 (since only three decimal places are retained, the number is shown as 0.000 in Table 4), which indicates that the water-saving irrigation projects focus more on the long-term social benefits. The short-term rate of return is found to be not that important, which is more in line with the approach of the current engineering practices. This point is taken into account in the X9 and X10 indicators of the three methods, which all give larger weight to the benefit coverage and to the ease of use for the farmers.

#### 3.1.2. Decision-Making Result of the Alternatives in Example 1

The MEM established in Section 2 is utilized to make decisions on water-saving irrigation project schemes. The score of each scheme is shown in Table 5, and the decision results are shown in Table 6. Table 5 also lists the decision results of the non-negative matrix factorization method (NMF for short) [36], the set pair analysis method (SPA for short) [37], the projection pursuit method (PP for short) [33], and the analytic hierarchy process (AHP for short) [23] methods.

The scores of each scheme in Table 5 show that the scores of the AHP schemes are very close to each other, around 0.7, while the difference between S3 and S4 is only 0.003, which is particularly easy to change in regard to the ranking, due to the calculation errors in the practical applications. In the SPA method, the score results of S1 and S2, as well as S3 and S4, are also very close. The score of S2 is nearly double that of S4. By comparing the index values of S2 and S4 in Table 1, it can be found that the two schemes should not have such a huge score gap. The score difference between S2 and S4 in the PP method is only 0.001, indicating that it is difficult for the PP method to distinguish between S2 and S4, thus this is the deficiency of the PP method. Different from the above three methods, the score results of the MEM and the NMF method have a useful distinction, and they can accurately distinguish the difference between the S2 and S4 schemes. As such, this is suitable for application in the teaching of water-saving irrigation project scheme decisions.

Table 6 shows that the scheme ranking results of the MEM is exactly the same as those of the other four methods, which indicate that the MEM is feasible for uncertain scheme decision-making. This is because the score values of each alternative in Example 1, and thus the ranking order of their corresponding alternatives, is S1**ϕ** S2**ϕ** S4**ϕ** S3 (here, **ϕ** denotes the “preferred to” relation). Therefore, S1 is the best option according to which water-saving irrigation schemes is better.

The decision-making results of Example 1 show that, compared with other methods, the MEM has better applicability and is more suitable to be applied in the teaching of water engineering economics. This will be particularly useful for students to master more reliable decision analysis methods, focus on learning decision indicator systems, and save time in decision-making method selection.

### 3.2. Example 2

#### 3.2.1. Calculation Result of Index Weight in Example 2

**Figure 3:**
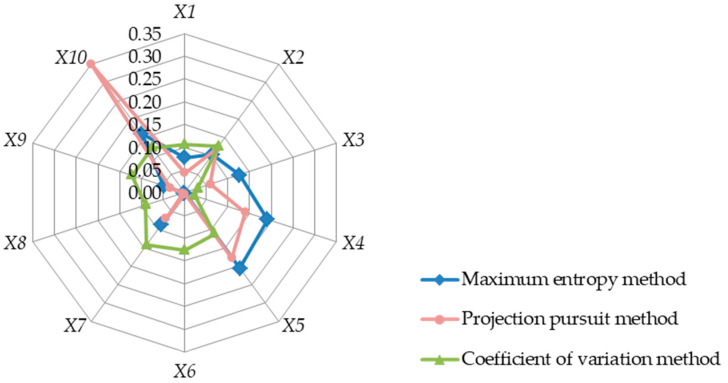
Radar map of the index weights of each method for Example 2.

The genetic algorithm is used to calculate the weight *w_j_* of each indicator in Table 2, according to the process in Figure 1 and which is shown in Table 7. The radar map of each indicator is shown in Figure 3. Same as Example 1, Table 7 also lists the index weight that was calculated by us, according to the various formulas in the PP and CV methods.

Table 7 shows that the PP method still has abnormally large index weight values, such as the index X10 (EEID) weight of 0.349, while the weight of the other indexes is too small. Such weight distribution produces great shortcomings in decision-making problems, which are easy to cause when the emphasis is on individual indicators and the role of other indicators is ignored. From Figure 3, it can be seen that the indicators with huge weight in the CV method are located in the lower left corner, while those with a huge weight in the PP method and the MEM are located in the upper right corner. By analyzing the meanings of indicators in Table 2, it can be seen that for water supply schemes, the investment in water supply schemes and their impact on flood control are more important, while the utilization of the original projects is slightly less important. Therefore, it is reasonable for the MEM and the PP methods to calculate the weight of the X6 index as 0.00044 (0.000 as shown in Table 7) and 0.003, respectively. Meanwhile, the CV calculates it as the second most important index after the water intake condition, which is obviously not in line with the actual situation. Similarly, it is not reasonable for the CV method to calculate the X7 index inlet condition as the most important index.

#### 3.2.2. Decision-Making Result of the Alternatives in Example 2

The MEM proposed in Section 2 is utilized to make decisions on urban water supply schemes. The score of each scheme is shown in Table 5, and the decision results are shown in Table 6. Table 5 also lists the calculation results of the NMF [36] and the grey relational degree method (GRD for short) [24]. For the convenience of comparison, we also calculated the decision result of Example 2 according to the PP method in the literature [33,34], which is also included in Table 8. Since the index weight of the SPA and AHP methods in the decision case of Example 2 cannot be obtained, the calculation of these two methods is not carried out in Example 2.

Table 8 shows that the score of the GRD methods are especially close to each other, with a maximum of 0.693 and a minimum of 0.550, which makes it particularly inconvenient for scheme decision-making. The overall differentiation of the PP score is better than the GRD score, but certain scores are too concentrated—such as in S2, S3, and S5—which is not convenient for decision-makers to make reasonable decisions. The score distribution of the NMF was better than that of the GRD and the PP methods on the whole, but S3 and S5 were also incredibly close, and the difference between the two scores was only 0.017. When observing the score results of the MEM, the score results of each scheme were well differentiated, and there was no indistinguishable situation between the schemes. For S2 and S3, which are very close in the GRD method, the MEM obtained S2 = 1.704 and S3 = 1.806, respectively, while for S1 and S6 in the GRD, the MEM obtained S1 = 0.930 and S6 = 0.851, indicating that the MEM is obviously superior to the GRD. For the three results of the PP method—which are very close to the score obtained in S2, S3, and S5—the results S2 = 1.704, S3 = 1.806, and S5 = 1.830 were obtained by the MEM, indicating that the MEM was significantly superior to the PP method. The MEM also shows a good differentiation between S3 and S5, which are very close to each other in the NMF. This result shows that the MEM, as the decision-making method of an engineering project scheme, is more suitable for beginners who are undertaking a water engineering economics course.

Table 9 shows the scores that are different from Example 1—i.e., where the sorting results of all methods are consistent; whereas, the sorting results of each method in Example 2 differ greatly. The four methods of Table 9 all take S4 as the optimal scheme, which indicates that the Gangli full open channel scheme S4 is the most appropriate in the Longhu Yellow Water Supply Diversion project of Zhengzhou. In general, the MEM, NMF, and PP methods all regard S1 and S6 as the inferior scheme and S2, S3, and S5 as the superior scheme, which is contrary to the results obtained by the GRD method. By comparing the index values of the S3 scheme and other schemes in Table 2, it is evidently not true that the GRD regards S3 as the worst scheme. Among the 10 indicators, 6 indicators of the S1 scheme are better than S6, and the X1 index of the S1 scheme is much shorter than that of the S6 scheme, which has huge economic benefits for reducing the operation cost of the water supply pipeline. Therefore, it is reasonable for both the MEM and NMF to consider S1 as superior to S6. Based on the above results and the analysis of Example 2, it can be seen that the MEM is feasible for water supply engineering scheme decision-making as the ranking results are more reasonable, which renders it particularly suitable for application in the teaching of water engineering economics.

### 3.3. Example 3

#### 3.3.1. Calculation Result of Index Weight in Example 3

**Figure 4:**
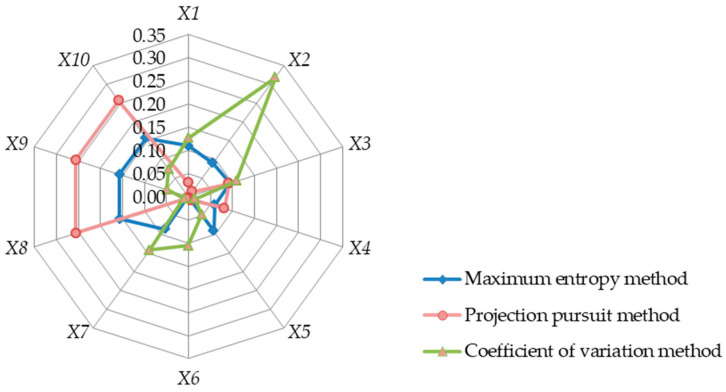
Radar map of the index weights of each method for Example 3.

The genetic algorithm was used to calculate the weight *w_j_* of each indicator in Table 3 according to the process in Figure 1, as shown in Table 10. The radar map of each indicator is illustrated in Figure 4. In Table 10, we also list the weights that were calculated by using the formulas utilized in the PP and CV methods.

Figure 4 shows the inverse distribution of the weight results of the CV, MEM, and PP methods. Table 10 shows that the CV method assigns the weight of index X2 to 0.318, while the weight of the other indicators is too small. This result shows that the CV method tends to favor industrial water use and ignores other aspects of water use in the context of water resources allocation, which is evidently inconsistent with engineering practice. In addition, the CV assigns the second most important weight to X7 sewage reuse. In fact, sewage reuse can only serve as a supplement to water resources. If X7 is given more prominent weight, the overall efficiency of water resources will be ignored.

The distribution of the weight results of the MEM and PP methods are similar, both of which are divided into two categories. One is for X8, and also X9 and X10, which have a large weight; the other indexes are relatively small. This indicates that both the MEM and PP methods indicate that improving the efficiency of water resources use is the most important index for water resources allocation, which is related to the low efficiency of industrial water use and agricultural water use in China.

#### 3.3.2. Decision-Making Result of the Alternatives in Example 3

The MEM that was established in Section 2 was utilized to make decisions on urban water supply schemes. The scores of each scheme are shown in Table 11, and the decision results are shown in Table 12. In Table 11, the calculation results of the NMF [36], the fuzzy optimization model (FOM for short) [25], and the back propagation artificial neural network (BPANN for short) [25] are also listed. For the convenience of comparison, we also calculated the decision result of Example 3 according to the PP method [33,34] in Example 1, which is also listed in Table 11.

From the perspective of differentiation regarding the decision results, the score results of the FOM and BP methods in Table 11 are not as distinguishable as those of the other three methods. The score results of S3 and S4 in the PP method are both 0.5; moreover, the differentiation is not particularly good. Both the MEM and NMF methods can clearly distinguish water resource allocation schemes, and the differentiation degree of the MEM is slightly better than that of the NMF method.

Table 12 shows that the ranking results of the decision schemes of all methods are generally consistent. It can be seen that S6, S5, S4, S3, S2, and S1 are all inferior water resources allocation schemes, and that the differences mainly lie in the ranking of S8 and S7. The FOM and PP methods all judged S7 to be superior to S8, while the NMF, BP, and MEM methods judged S8 to be superior to S7. By analyzing the composition of these two schemes, it can be found that the only difference between S7 and S8 is in terms of “current seawater utilization” and “increased seawater utilization”. S7 focuses on “current seawater utilization”, while S8 focuses on “increasing seawater utilization.” Although adding sea water consumption into the S8 scheme will increase the investment in water conservancy projects, part of the surplus industrial water can be used as ecological water for urban rivers and lakes [25], which is more consistent with the actual situation. Overall, the S8 scheme is more reasonable. In the decision-making process of the FOM method, the weight of each indicator needs to be determined subjectively, which will cause the weight distribution and decision ranking to be affected by subjective factors, such as expert experience, thus resulting in the result that S7 is superior to S8. The main reason why S7 is superior to S8, as determined by the PP method, is that PP method assigns a weight difference between S8 and S7, among which the weight of S8 is 0.256 and S7 is 0.005. Therefore, the score result of such a scheme decision demonstrates that S7 is superior to S8.

Based on the above analysis of Example 3, it can be seen that the MEM has also been well applied in water resources allocation decision-making, with reasonable decision-making results and high differentiation. These qualities render it as being easier to adopt in the teaching practice of water engineering economics courses.

### 3.4. Discussions

The results of the three examples corresponding to the objectives proposed in the introduction are discussed as follows.

#### 3.4.1. The Applicability Characteristics

In the case study section, we apply the MEM to carry out decision-making research on three water engineering example problems, respectively. These examples have different decision index systems and solve different problems. However, the MEM was well applied in these cases, and the decision results are consistent with other existing methods on the whole, which shows the wide applicability of the MEM. This is mainly because we abstract the actual water engineering decision problem into a mathematical problem through the four steps in Section 2.1 when constructing the MEM, such that it can be implemented in all kinds of water engineering decision problems.

It should be noted that the MEM, as is the case in other objective decision-making methods such as the PP method, relies on the decision matrix being established in advance. Therefore, whether the MEM can be applied to other decision-making problems regarding water engineering schemes mainly depends on the following three points. First, it is evident that the alternatives in the decision matrix are reasonable. Second, the attribute values of the alternative scheme are real numbers; for the attribute values in the form of random numbers, interval numbers, fuzzy numbers, and other variables, the MEM needs to be further expanded before it can be used, which is one of the limitations of the MEM established in this paper. Third, the number of alternative schemes should not be too small, it is suggested that they should be more than or equal to 4. According to the standard deviation formula of Equation (7), it can be found that investigating not enough alternative schemes will lead to the statistical characteristics of the score values to deviate from reality. Therefore, we make this suggestion when applying the MEM, which is the second limitation of the MEM that is established in this paper.

Although the MEM has excellent applicability, the above three assumptions and limitations need to be noted when applied.

#### 3.4.2. The Differentiation Characteristics

Table 5, Table 8 and Table 11, respectively, show the score values of the decision results of the three cases. These tables all show that the differentiation degree of the MEM is close to that of the NMF and PP methods, showing a better differentiation in terms of decision results, while the scores of each scheme of the AHP, GRD, and FOM methods are very close, which render them as not easy to use in order to make a correct decision. From the analysis of the principle of the method, we can see that the MEM takes the standard deviation of the score value of each scheme as the objective function, and directly constructs the optimization model with the goal of the maximum differentiation, which is the main reason for the good differentiation of the decision results of the MEM. Through this processing, we transform the multi-attribute decision problem into an optimization problem with the attribute weight as the optimization variable, which can be optimized by the mature genetic algorithm. Beginners who are learning how to best apply this method can directly use MATLAB, Python, or other software in the genetic algorithm toolbox calculation.

#### 3.4.3. The Objective Characteristics

Compared to the PP and CV methods, the MEM does not add subjective information in the weight calculation process of the three examples; therefore, the weight that is obtained is objective. However, there are also significant differences between these objective methods. In terms of the weight calculation principle, the MEM is similar to the PP method, both of which indirectly make use of the difference between the attribute values of the various alternative schemes, which are similar to statistical standard deviations. It is different from the CV method in that it directly uses the standard deviation and mean values of the attribute. Therefore, it can be seen from the weight calculation results of the three examples that the weight result of the MEM is close to that of the PP method; however, it differs greatly from those obtained by the CV method. This shows that in the same three weight calculation methods when using objective information, the weight calculation results are also different.

As for the additional addition of information, namely information gain, the MEM adopts the objective function of the maximum entropy of weight, such that the weight value obtained is more objective from the perspective of information gain. As can be seen from the results of Figure 2, Figure 3 and Figure 4, there is no outstanding abnormal weight in the weights obtained by the MEM. This phenomenon shows that, without adding supplementary information, the entropy of the weight is at the maximum and that this objective function makes the weight as close to a uniform distribution as possible. This is quite consistent with the lack of subjective experience that beginners possess in water engineering economics courses. As such, it is suitable to adopt the MEM in the teaching of water engineering economics courses.

#### 3.4.4. The Rationality Characteristics

When the MEM was applied to solve the first example, the preference ranking results of the alternatives were the same as those of the other methods. For the other two examples, the MEM also showed consistent sorting results with other existing methods on the whole. Alternative preference ranking results of these three different data sets all show that the MEM is reasonable.

The rationality of the decision result of the MEM is guaranteed by the objective function Equation (9) constructed. On the one hand, the maximum entropy of attribute weight ensures that no abnormal information will appear in regard to the objective weight obtained, which is especially evident in the weight calculation that is performed via the CV method. Only when the result of attribute weight calculation is reasonable will the result of alternative scheme preference ranking be reasonable. On the other hand, the score values of each alternative scheme should be differentiated as much as possible, which can ensure that the ranking results according to preference will not change due to calculation errors and various uncertainties, thus showing the robustness of the ranking results.

Through the above discussion, it can be found that the proposed MEM has shown widespread applicability, rationality, discriminability, and objectivity in the three examples, thereby achieving the main objective of this research that was proposed in the introduction section.

## 4. Conclusions

From Examples 1–3, we conclude that the proposed MEM was successfully applied to solve decision-making problems in the water engineering economics course. Meanwhile, the other existing methods demonstrated various shortcomings. Therefore, our method is superior and more effective for solving decision-making problems.

The key contributions of the work can be briefly summarized below.

(1) We put forward a maximum-entropy-based decision-making method for the purposes of teaching practice in the course of water engineering economics courses. The proposed MEM considers the uncertainty of weight in the decision-making process, then integrates weight calculation and decision scheme score calculations into an objective function. Compared to the traditional two-stage weight and decision method, this calculation process is more convenient. Furthermore, it is convenient for decision-makers to learn and master, and it is easy to popularize and apply.
(2) In terms of the score value in regard to the decision result, the MEM shows outstanding applicability in terms of the differentiation and rationality of the decision scheme. This is well illustrated in all three examples. However, other comparison methods have some shortcomings, such as in them being difficult to use in distinguishing decision results and unreasonable decision schemes.
(3) In terms of weight calculation, the MEM shows robust calculation results in the three examples, while the other methods have more or less shortcomings. Some show obvious abnormal index weights, some index weights are inconsistent with the reality, and some obtain a weight distribution that is evidently different from the other methods.
(4) To demonstrate the feasibility, reliability, and effectiveness of the proposed approaches, three examples were given to compute their results based on the proposed MEM. These three examples are typical water project scheme decision problems. The case study in this paper also provides a good illustration of the application of the MEM in similar scheme decision problems.

To sum up, the four primary research objectives of this paper proposed in the introduction section have been achieved. However, it should be noted that a scheme decision is only a part of the economic content of water engineering, and that the MEM is only a tool in the economic toolbox of water engineering. While learning in water engineering economics courses, it is also essential to master a series of basic concepts regarding water engineering economics, as well as the construction of a decision index system and the formulation of decision schemes. 

The main assumption of this study is that the problem to be solved is decision-able, which has to be controlled by the decision-makers in the scheme construction stage. In addition, the limitation of this study is that each alternative attribute value variable needs to be a real number and that the number of alternative schemes should not be too small. For example, if there are only two schemes, the weight calculated by the proposed MEM will have significant uncertainties. Therefore, we suggest using the proposed MEM only for decision problems where the number of alternatives is more important than or equal to 4. As for the further research of the MEM, we suggest taking the subjective weight of each decision indicator made by experts as the constraint conditions of the MEM, and then applying the genetic algorithm to solve the index weights and score values. The resulting decision results will not only reflect the rich experience of the decision experts, but also give full play to the advantages of the maximum entropy method in scheme differentiation and other aspects.

## Figures and Tables

**Table 1:** Evaluation index value of the water-saving irrigation schemes for Example 1.

No.	X1	X2	X3	X4	X5	X6	X7	X8	X9	X10	X11
S1	11.26	0.112	12.8	1.86	85.2	9.3	85	92	100	100	0.9
S2	9.2	0.128	13.2	2.10	91.6	9.1	92	90	90	80	0.8
S3	10.8	0.135	14.2	1.94	82.4	8.60	95	86	80	70	0.7
S4	9.6	0.142	11.5	1.65	83.2	9.2	90	82	100	90	0.8

In Table 1, X3: PP belongs to the smaller and better type of index, while the other indicators are of the bigger and better type.

**Table 2:** Evaluation index value of the water-supply schemes for Example 2.

No.	X1	X2	X3	X4	X5	X6	X7	X8	X9	X10
S1	4.51	36,300	1298.31	9.75	14.33	7.03	8.46	2.31	16.98	2.03
S2	16.43	12,065	1098.65	8.53	7.17	14.06	2.81	11.59	4.85	14.26
S3	15.34	12,968	889.21	8.62	7.17	11.71	2.81	6.95	7.27	10.19
S4	13.03	13,950	1215.66	8.72	3.58	3.87	19.73	11.59	9.7	18.33
S5	13.03	15,320	1285.76	9.12	3.58	2.34	19.73	6.95	12.13	6.11
S6	28.39	8054	1359.3	11.2	10.75	16.4	14.09	9.26	2.42	8.14

In the above table, X6: ROP, X7: SWIC, X9: WQGD, and X10: EEID all belong to the larger is better type, while the other indexes belong to the smaller is better type.

**Table 3:** Evaluation index value of the water supply schemes for Example 3.

No.	X1	X2	X3	X4	X5	X6	X7	X8	X9	X10
S1	29.69	28.79	45.36	1157.76	105.64	34.05	3.6	80	0.57	18
S2	26.26	11.19	45.36	1099.1	143.49	58.63	3.6	80	0.57	18
S3	21.77	16.22	35.05	1112.67	132.53	49.3	8.29	80	0.57	18
S4	18.34	0.00	35.05	1040.76	159.61	73.48	8.29	80	0.57	18
S5	22.28	21.94	31.11	1204.84	119.73	60.01	3.6	85	0.75	12
S6	17.82	3.15	31.11	1145.05	159.11	84.19	3.6	85	0.75	12
S7	11.97	8.52	18.12	1168.91	149.85	80.86	8.29	85	0.75	12
S8	7.5	0.00	18.12	1111.11	159.61	99.04	8.29	85	0.75	12

In the index system of an optimal water resources allocation scheme in Table 3, X1: RWLR, X2: IWLR, X3: AWLR, X6: IWP, and X10: UWSPLR all belong to the smaller is better type, while the other indicators belong to the bigger is better type.

**Table 4:** Index weights of each comparison methods for Example 1.

Methods	X1	X2	X3	X4	X5	X6	X7	X8	X9	X10
PP	0.029	0.002	0.092	0.004	0.074	0.223	0.002	0.135	0.128	0.129
CV	0.104	0.108	0.094	0.108	0.053	0.037	0.051	0.055	0.113	0.165
Proposed MEM	0.036	0.000	0.098	0.048	0.093	0.094	0.048	0.108	0.160	0.154

**Table 5:** Score values of each the comparison methods for Example 1.

Alternatives	NMF	SPA	PP	AHP	MEM
S1	2.326	0.247	2.423	0.747	2.375
S2	1.890	0.216	1.643	0.730	1.814
S3	0.950	0.019	0.399	0.713	0.648
S4	1.834	0.104	1.642	0.716	1.620

**Table 6:** Comparative analysis for Example 1.

Methods	Ranking
NMF, Xie, and Huang [36]	S1**ϕ** S2**ϕ** S4**ϕ** S3
SPA and Gao [37]	S1**ϕ** S2**ϕ** S4**ϕ** S3
PP, Jin, and Liu [33]	S1**ϕ** S2**ϕ** S4**ϕ** S3
AHP and Zhang [23]	S1**ϕ** S2**ϕ** S4**ϕ** S3
Proposed MEM	S1**ϕ** S2**ϕ** S4**ϕ** S3

**Table 7:** Index weights of each comparison methods for Example 2.

Methods	X1	X2	X3	X4	X5	X6	X7	X8	X9	X10
PP	0.044	0.120	0.060	0.141	0.177	0.003	0.071	0.004	0.033	0.349
CV	0.106	0.126	0.030	0.023	0.112	0.127	0.143	0.090	0.122	0.122
Proposed MEM	0.077	0.103	0.126	0.191	0.206	0.000	0.087	0.002	0.048	0.160

**Table 8:** Score values of each of the comparison methods for Example 2.

Alternatives	NMF	GRD	PP	MEM
S1	1.210	0.577	0.795	0.930
S2	1.810	0.557	1.708	1.704
S3	1.917	0.550	1.709	1.806
S4	2.134	0.693	2.205	2.207
S5	1.900	0.633	1.709	1.830
S6	1.150	0.574	0.951	0.851

**Table 9:** Comparative analysis for Example 2.

Methods	Ranking
NMF, Xie, and Huang [36]	S4**ϕ** S3**ϕ** S5**ϕ** S2**ϕ** S1**ϕ** S6
GRD, Zhao, and Guo [24]	S4**ϕ** S5**ϕ** S1**ϕ** S6**ϕ** S2**ϕ** S3
PP	S4**ϕ** S5, S3**ϕ** S2**ϕ** S6**ϕ** S1
Proposed MEM	S4**ϕ** S5**ϕ** S3**ϕ** S2**ϕ** S1**ϕ** S6

**Table 10:** Index weights of each comparison methods for Example 3.

Methods	X1	X2	X3	X4	X5	X6	X7	X8	X9	X10
PP	0.030	0.014	0.092	0.082	0.009	0.002	0.005	0.256	0.256	0.256
CV	0.127	0.318	0.109	0.015	0.049	0.106	0.143	0.011	0.050	0.073
Proposed MEM	0.109	0.089	0.094	0.059	0.092	0.000	0.087	0.156	0.156	0.156

**Table 11:** Score values of each of the comparison methods for Example 3.

Alternatives	NMF	FOM	BPANN	PP	MEM
S1	0.366	0.018	0.200	0.244	0.180
S2	0.718	0.122	0.235	0.291	0.537
S3	1.097	0.253	0.311	0.500	0.922
S4	1.337	0.346	0.328	0.500	1.185
S5	1.875	0.666	0.683	2.096	1.855
S6	2.265	0.817	0.739	2.157	2.247
S7	2.702	0.972	0.808	2.422	2.703
S8	2.785	0.945	0.818	2.397	2.825

**Table 12:** Comparative analysis for Example 3.

Methods	Ranking
NMF, Xie, and Huang [36]	S8**ϕ** S7**ϕ** S6**ϕ** S5**ϕ** S4**ϕ** S3**ϕ** S2**ϕ** S1
FOM, Yang [25]	S7**ϕ** S8**ϕ** S6**ϕ** S5**ϕ** S4**ϕ** S3**ϕ** S2**ϕ** S1
BPANN, Yang [25]	S8**ϕ** S7**ϕ** S6**ϕ** S5**ϕ** S4**ϕ** S3**ϕ** S2**ϕ** S1
PP	S7**ϕ** S8**ϕ** S6**ϕ** S5**ϕ** S3, S4**ϕ** S2**ϕ** S1
Proposed MEM	S8**ϕ** S7**ϕ** S6**ϕ** S5**ϕ** S4**ϕ** S3**ϕ** S2**ϕ** S1

## Data Availability

The raw data supporting the conclusion of this article will be made available by the authors, without undue reservation.
